# Twentieth Semi-Annual Meeting of the Brazos Valley Medical Association

**Published:** 1906-01

**Authors:** W. B. Briggs

**Affiliations:** Secretary


### Twentieth Semi-Annual Meeting of the Brazos Valley Medical Association.

The Brazos Valley Medical Association convened at Rockdale, November 14th and 15th, and was called to order by the president, Dr. H. T. Coultor. Most interesting papers were read by Drs. J. P. Oliver, Caldwell; J. W. Torbett, Marlin; A. G. Krueger, Caldwell; G. H. Moody, San Antonio; E. N. Shaw, Cameron; H. N. Graves, Georgetown; Daniel Parker, Calvert; I. P. Sessions, Rockdale; H. E. Baine, Lexington; Lee and Ramsell, Thorndale; A. S. Epperson, Cameron; A. C. Scott, Temple; W. A. Harper, Austin, and Dr. Wm. Keiller, of Galveston Medical College, who had the kindness to bring his beautifully colored models to aid in illustrating his two papers: "Surgical Anatomy of the Axillary Lymph Glands in Their Relation to Mammary Cancer," and "Anatomy of Surgical Hernia." A rising vote was tendered Professor Keiller.

At night a most sumptuous banquet was tendered the Association. Resolutions were passed endorsing and complimenting Dr. Geo. R. Tabor highly for the stand taken by him in protecting Texas by his strict quarantine. The great question: "Shall the Brazos Valley Medical Association re-organize and become part of the Brazos Valley District Medical Association?" was discussed. Speaking, pro and con, was progressing when Dr. J. W. Hudson, of Milano, moved to postpone consideration of this subject for five years.

The next meeting will take place at Hearne the second Tuesday and Wednesday in May next.

W. B. Briggs,
Secretary.

---

*Dr. F. E. Daniel, Austin, Texas.*

Dear Doctor: Enclosed please find money order for one dollar for the "Red Back" one more year. Here's for Dr. McCormack, State, district, and county associations; and three *big* cheers for the Texas Medical Journal against quacks, etc.

Yours until we meet again.

Respectfully and fraternally, etc.,

Joshua, Texas, December 8, 1905. J. M. Townes, M. D.

[Twenty-first consecutive year.—Ed.]

